# Preparation and anti-triple-negative breast cancer cell effect of a nanoparticle for the codelivery of paclitaxel and gemcitabine

**DOI:** 10.1186/s11671-023-03899-1

**Published:** 2023-09-21

**Authors:** Fan Yang, Zehui Fan, Lixia Zhang, Yanjuan He, Run Hu, Jinkun Xiang, Shiyang Fu, Guowei Wang, Jianlong Wang, Xiaojun Tao, Pan Zhang

**Affiliations:** 1 Key Laboratory of Study and Discovery of Small Targeted Molecules of Hunan Province and Department of Pharmacy, School of Medicine, Hunan Normal University, 371 Tongzipo Road, Changsha, 410013 Hunan China; 2 Department of Pediatrics, The Fourth Hospital of Changsha, 70 Lushan Road, Changsha, 410006 Hunan China; 3 Department of Spine Surgery and Department of Infection, The Third Xiangya Hospital of Central South University, 138 Tongzipo Road, Changsha, 410013 Hunan China

**Keywords:** Triple-negative breast cancer, Paclitaxel, Gemcitabine, Nanodrug delivery system

## Abstract

Amphiphilic polymers (HA-ANI) were prepared by grafting hyaluronic acid (HA) and 6-(2-nitroimidazole)hexylamine (ANI) and then self-assemble in water to form nanoparticles (NPs) that could be loaded with paclitaxel (PTX) and gemcitabine (GEM) by dialysis. Infrared spectroscopy and ^1^H-NMR indicated the successful synthesis of HA-ANI. Three different ratios of NPs were prepared by adjusting the ratios of hydrophilic and hydrophobic materials, and the particle size decreased as the ratio of hydrophilic materials increased. When HA:ANI = 2.0:1, the nanoparticles had the smallest size distribution, good stability and near spherical shape and had high drug loading and encapsulation rates. In vitro release experiments revealed that NADPH could accelerate the drug release from NPs. Cellular uptake rate reached 86.50% at 6 h. The toxic effect of dual drug-loaded nanoparticles (P/G NPs) on MDA-MB-231 cells at 48 h was stronger than that of the free drug. The AO/EB double-staining assay revealed that a large number of late apoptotic cells appeared in the P/G NPs group, and the degree of cell damage was significantly stronger than that of the free drug group. In the cell migration assay, the 24 h-cell migration rate of the P/G NPs group was 5.99%, which was much lower than that of the free group (13.87% and 17.00%). In conclusion, MDA-MB-231 cells could effectively take up P/G NPs, while the introduction of the nano-codelivery system could significantly enhance the toxicity of the drug to MDA-MB-231 cells as well as the migration inhibition effect.

## Introduction

Triple-negative breast cancer (TNBC) is a highly metastatic, heterogeneous disease with a poor prognosis and high recurrence rate up to five years after treatment compared to non-TNBC cases [1, 2]. There is a lack of targeted drugs due to low expression of the three major receptors (ER, PR and HER-2) [3]. Systemic chemotherapy remains the main treatment modality for patients with TNBCs [4, 5], and drug resistance often occurs after chemotherapy [6, 7]. There is an urgent clinical need to develop new chemotherapy strategies that target resistance and potentiation.

Paclitaxel [8] is the first-line drug for the conventional treatment of triple-negative breast cancer and mainly inhibits mitosis and causes apoptosis in cancer cells by stabilizing and enhancing the polymerization of microtubule proteins and preventing microtubule depolymerization [9, 10]. Conventional paclitaxel drugs are bound by cosolvents as well as liposomal phospholayers, which are slow-releasing, toxic, and prone to causing allergic reactions and therefore require preemptive anti-allergic treatment prior to administration, as well as long infusion times, limiting their application to some extent [11, 12]. Gemcitabine (GEM) [13] is a pyrimidine antitumor drug whose main metabolite penetrates into DNA intracellularly, interferes with DNA synthesis, and inhibits nucleotide reductase. The results of several clinical trials have shown that its combination with albumin paclitaxel can achieve better therapeutic effects [14–16]; however, neuropathy as well as gastrointestinal reactions are still more pronounced [17]. Therefore, it is important to enhance the anti-TNBC effect by combining paclitaxel and gemcitabine through different pathways and anticancer mechanisms and to develop a novel delivery system to improve its efficacy and reduce toxic side effects to improve the therapeutic effect.

Nanodelivery systems have good biocompatibility, low toxicity, targeting, and controlled release properties, which offer good prospects in cancer therapy [18, 19]. More importantly, nanocarrier-based antitumor drug delivery systems clearly show the potential to overcome the problems associated with conventional chemotherapy [20]. Nanodrug codelivery systems include at least two anticancer drugs with different physicochemical and pharmacological properties loaded into a single delivery system, solving the problems associated with the differences in the biodistribution and transport mechanisms of the two drugs [21]. Nanoparticles with different surface properties can be prepared by adjusting the ratio of different components in the carrier material as well as the ratio of drug to carrier while optimizing the drug loading and encapsulation rate, with a higher loading capacity favoring a better therapeutic effect. Passive targeting is achieved by modulating the size and surface properties of nanoparticles to promote their selective accumulation in tumors through the EPR effect [22]. Active targeting can be achieved by modifying nanomaterials with high-affinity ligands, such as HA [23], folic acid [24], and mannose [25]. In addition, nanoparticles can effectively respond to the stimulation of the tumor microenvironment (e.g., low pH [26], high glutathione (GSH) [27]and reactive oxygen species (ROS) levels [28]) and release drugs in a controlled manner, thus killing tumor cells and protecting normal tissues from damage.

**Fig. 1:**
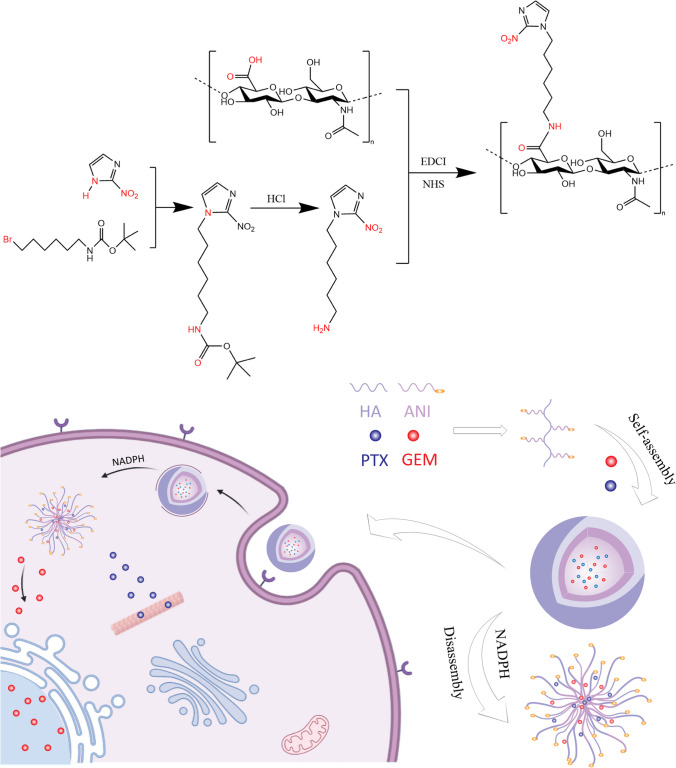
Schematic diagram of the preparation of P/G NPs and their application in combination chemotherapy

In this study, amphiphilic polymers were prepared by grafting hyaluronic acid (HA) and 6-(2-nitroimidazolyl)hexylamine (ANI). The resulting HA-ANI self-assembled in water to form nanoparticles that could be coloaded with the chemotherapeutic drugs PTX and GEM for combined chemotherapy. As shown in Fig. 1, nanoparticles can preferentially accumulate at tumor sites through the EPR effect and enter tumor cells through CD44 receptor-mediated endocytosis. Inside the tumor cells, the nanoparticles rapidly dissociate under the action of the bioreductant NADPH, releasing PTX and GEM into the cytoplasm to exert antitumor effects. A series of in vitro experiments were conducted to demonstrate the feasibility and advantages of the nanodrug delivery system.

## Materials and methods

### Materials

PTX, 2-nitroimidazole and N-Boc-6-bromohexylamine were purchased from Aladdin Reagent (Shanghai) Co., Ltd. GEM, N-hydroxysuccinimide and N-(3-dimethylaminopropyl)-N′-ethylcarbodiimide hydrochloride were purchased from Maclean's Biochemical Technology Co., Ltd. Dimethyl sulfoxide was purchased from Sinopharm Reagent (Shanghai) Co., Ltd. Phosphate buffered salt solution (PBS) and DMEM were purchased from Gibco (USA). Fetal bovine serum was purchased from BI.

MDA-MB-231 cells were obtained from Prof. Xiyun Deng's group at the School of Medicine, Hunan Normal University in September 2021.

### Synthesis of ANI

2-Nitroimidazole (150 mg; 1.33 mmol) and K_2_CO_3_ (280 mg; 2.03 mmol) were dissolved in dimethylformamide (DMF). Then, N-Boc-6-bromohexylamine (390 mg; 1.39 mmol) was added to the DMF solution, and the reaction was stirred at 80 °C for 4 h. The solid impurities were removed by filtration and washed with methanol. The solid product was obtained by washing with methanol and evaporating the residual solvent. The above solid was suspended in deionized water and extracted with ethyl acetate, the organic layer was collected and the BOC-protected 6-(2-nitroimidazolyl)hexylamine (QANI) was obtained by rotary evaporator. This product was redissolved in methanol, and 5 mL of 1.25 M HCl was added to the methanol solution and stirred at room temperature for 24 h. Afterward, the product ANI was obtained by removing the solvent from the reaction mixture using a rotary evaporator.

### Synthesis of HA-ANI

60 mg of HA (molecular weight of approximately 30 kDa) were dissolved in water, to which N-(3-dimethylaminopropyl)-N'-ethylcarbodiimide hydrochloride (EDCI) (107.35 mg; 0.56 mmol) and N-hydroxysuccinimide (NHS) (64.40 mg; 0.56 mmol) were added and stirred at room temperature for 1 h. ANI (30 mg; 0.14 mmol) was added to the mixture, and the reaction was carried out at room temperature for 24 h. The reaction mixture was transferred to a dialysis bag (MW = 3000) for dialysis, and the product HA-ANI was obtained by lyophilization after prefreezing at -20 ℃.

### Preparation of P/G NPs

Approximately, 10 mg of HA-ANI polymer was dissolved in DMSO and stirred at 40 °C for 2 h. Approximately, 5 mg of PTX and 2 mg of GEM were predissolved in appropriate amounts of DMSO and then mixed with HA-ANI solution and stirred at 40 °C for 4 h. The liquid mixture was then transferred to a dialysis bag (MW = 3000) and dialyzed with deionized water to completely remove DMSO. After prefreezing at -20 ℃, the lyophilization was performed in a freeze-dryer to obtain P/G NPs. HA-ANI NPs and HA-ANI@Cy5 NPs were prepared by the same method.

### Characterization of P/G NPs

The structures of HA, ANI and HA-ANI were characterized by infrared spectroscopy and nuclear magnetic resonance hydrogen spectroscopy (^1^H-NMR); the size, potential and polymer dispersity index (PDI) of the nanoparticles were detected by dynamic light scattering (DLS); and the morphology of the nanoparticles was observed by transmission electron microscopy.

### Stability of P/G NPs

The P/G NPs were stored at room temperature and monitored for changes in particle size, potential, and PDI on days 1, 3, 5, 7, 14, and 21.

### Drug loading capacity and encapsulation efficiency

Five milligrams of paclitaxel and gemcitabine were dissolved in 25 mL of DMSO, respectively, and prepared into a standard stock solution of 200 µg/mL. Then, a series of solutions with different concentrations were prepared using the dilution method to draw the standard curve. The absorbance of P/G NPs was measured by UV spectrophotometry, and the drug loading capacity and encapsulation efficiency were calculated using the standard curve equation.

$${\text{Drug}}\;{\text{loading}}\;{\text{capacity}}\left[ {{\text{LC}}\left( \% \right)} \right] = \frac{{W_{{{\text{loaded}}}} }}{{W_{{{\text{P}}/{\text{G}}\;{\text{NPs}}}} }} \times 100\%$$

$${\text{Encapsulation}}\;{\text{efficiency}}\left[ {{\text{EE}}\left( {\text{\% }} \right)} \right] = \frac{{W_{{{\text{loaded}}}} }}{{W_{{{\text{added}}}} }} \times 100\%$$

### In vitro drug release

First, 1 mL of 1 mg/mL P/G nanoparticles was precisely measured into each dialysis bags (MW = 3000). The bags were then placed in pH 7.4 PBS and pH 7.4 PBS containing 1 mM NADPH, 1 mL of 1 mg/mL PTX and 1 mL of 1 mg/mL GEM was placed in dialysis bags (MW = 3000). The dialysis bags were then placed in pH 7.4 PBS and shaken at 37 °C and 100 rpm, protected from light. A 3 mL sample of release medium was taken at 0, 0.083, 0.25, 0.5, 1, 2, 4, 8, 12, 24, 36, 48, and 72 h, while the same volume of release medium was used to replenish the tube. A UV‒vis spectrophotometer was used to determine the release of PTX and GEM, and the release rates were calculated separately according to the following equations.

$$Q\left( \% \right) = \left( {C_{n} \times V + V_{n} \mathop \sum \limits_{t = 0}^{n} C_{i} } \right)/W*LC\left( \% \right)$$

*W* is the total weight of nanoparticles, *C*_*n*_ is the sample concentration at *T*_*n*_, *V* is the total volume of release medium, *V*_*n*_ is the sample volume, and *C*_*i*_ is the sample concentration at *T*_*i*_ (*i* = 0,0.083,0.25…… hours, *V*_0_, *C*_0_ is equal to 0).

### Cell culture

MDA-MB-231 cells were selected as the model cell line, and the cells were cultured in DMEM containing 10% FBS and 1% PS in an incubator at 37 ℃ and 5% CO_2_.

### Cell uptake

MDA-MB-231 cells at the exponential growth stage were seeded in 6-well plates (2 mL, 5 × 10^4^ cells/well) and incubated at 37 °C and 5% CO_2_ for 24 h until the cell wall fusion rate reached 80%. HA-ANI@Cy5 NP-treated cells were placed in a cell culture incubator at 37 °C and 5% CO_2_ for 2 h, 4 h and 6 h, and then, the culture medium was aspirated and discarded. The cells were washed gently with PBS 3 times and fixed with 4% paraformaldehyde for 15 min, after which they were stained with DAPI staining solution for 5 min, and images were taken under an inverted fluorescence microscope.

### In vitro cytotoxicity

Cytotoxicity studies were performed by the MTT method. MDA-MB-231 cells at the exponential growth stage were grown in 96-well plates (100 μL, 5000 cells/well) and incubated at 37 °C and 5% CO_2_ for 24 h until the cells were completely adhered to the wall. The cells were treated with serial concentrations of free PTX, free GEM, free PTX/GEM or P/G NPs and incubated in a cell incubator at 37 °C and 5% CO_2_ for 48 h. For the control group, an equal volume of medium was added to continue the culture. 48 h later, 20 µL of 5 mg/mL MTT solution was added to each well, and the culture was continued for 4 h. After careful aspiration and discarding of the culture solution, 150 µL of DMSO was added to each well, and the cell survival rate was calculated by determining the OD value of each well at 570 nm with a multifunctional enzyme marker after shaking for 15 min at room temperature.

### Cell migration

Cell migration was studied by scratch assay. MDA-MB-231 cells at the exponential growth phase were seeded in 6-well plates (2 mL, 1 × 10^5^ cells/well) and incubated at 37 °C with 5% CO_2_ for 24 h until the cell wall fusion rate reached 100%. Straight lines were drawn in the wells with a 10 μL pipette tip, and the scratched cells were rinsed with PBS, followed by the addition of serum-free DMEM containing P/G NPs (C_PTX_ = 0.16 μM; C_GEM_ = 0.057 μM), PTX (0.16 μM), GEM (0.057 μM), and PTX/GEM (C_PTX_ = 0.16 μM; C_GEM_ = 0.057 μM). The cells were incubated in a constant temperature incubator. Cell images were taken at 0, 6, 12 and 24 h.

### Cell apoptosis analysis

Apoptosis was studied by the AO/EB double-staining method. MDA-MB-231 cells at the exponential growth phase were seeded in 6-well plates (2 mL, 5 × 10^4^ cells/well) and incubated at 37 °C with 5% CO_2_ for 24 h. Then, 200 μl of P/G NPs (C_PTX_ = 0.16 μM; C_GEM_ = 0.057 μM), PTX (0.16 μM), GEM (0.057 μM), and PTX/GEM (C_PTX_ = 0.16 μM; C_GEM_ = 0.057 μM) was used to treat cells and the same volume of PBS was added to the control group. 48 h later, the cells were collected, the density was adjusted to 1 × 10^6^ cells/mL, the prepared AO/EB staining solution was added and incubated at room temperature for 15 min, and images were obtained by inverted fluorescence microscopy.

### Statistical analyses

Prism software was used for statistical significance analysis. The signal indicated a significant difference (*P < 0.01, **P < 0.005, ***P < 0.001, ****P < 0.0001).

## Results

### Preparation and characterization of P/G NPs

**Fig. 2:**
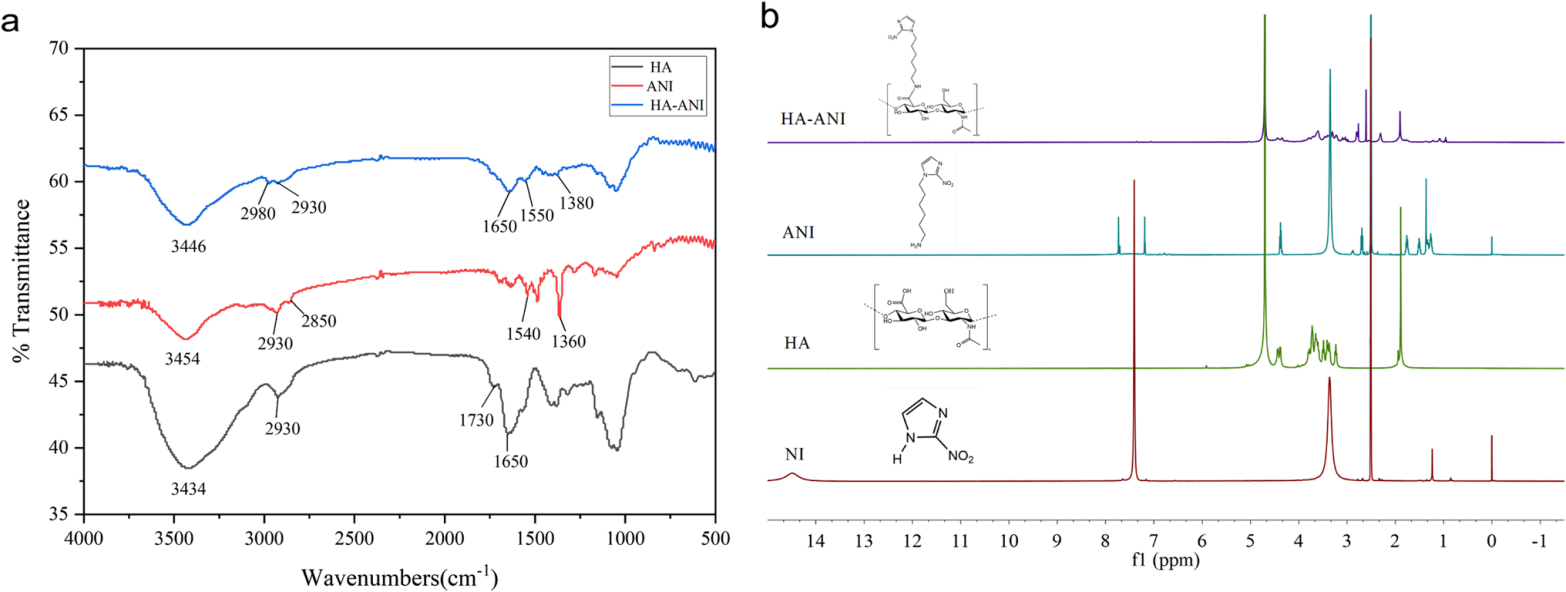
Structural characterization of HA-ANI polymers. **a** IR spectra of HA, ANI and HA-ANI. **b** NMR hydrogen spectra of NI, HA, ANI and HA-ANI

We grafted HA and ANI by an amide reaction and characterized the structures of NI, HA, ANI, and HA-ANI using infrared spectroscopy and nuclear magnetic resonance hydrogen spectroscopy (^1^H-NMR). In the IR spectrum (Fig. 2a), ANI had a stretching vibrational absorption of -NO_2_ at 1360 cm^−1^ and 1540 cm^−1^, and HA-ANI showed a stretching vibrational absorption of -NO_2_ at 1380 cm^−1^ and 1550 cm^−1^ due to the shift of conjugation to lower wavenumbers; moreover, HA had a stretching vibrational absorption of C=O of amide at 1650 cm^−1^ and 1730 cm^−1^, and the C=O stretching vibration absorption of carboxylic acid at 1730 cm^−1^ in HA-ANI disappeared, which showed the successful attachment of 6-(2-nitroimidazole)hexylamine to hyaluronic acid through the amide bond. In the ^1^H-NMR (Fig. 2b), the shielding effect of the heterocyclic ring in HA shifted the chemical shift of each hydrogen spectrum in HA-ANI upfield. All the above results suggested the successful synthesis of the HA-ANI polymer.

**Fig. 3:**
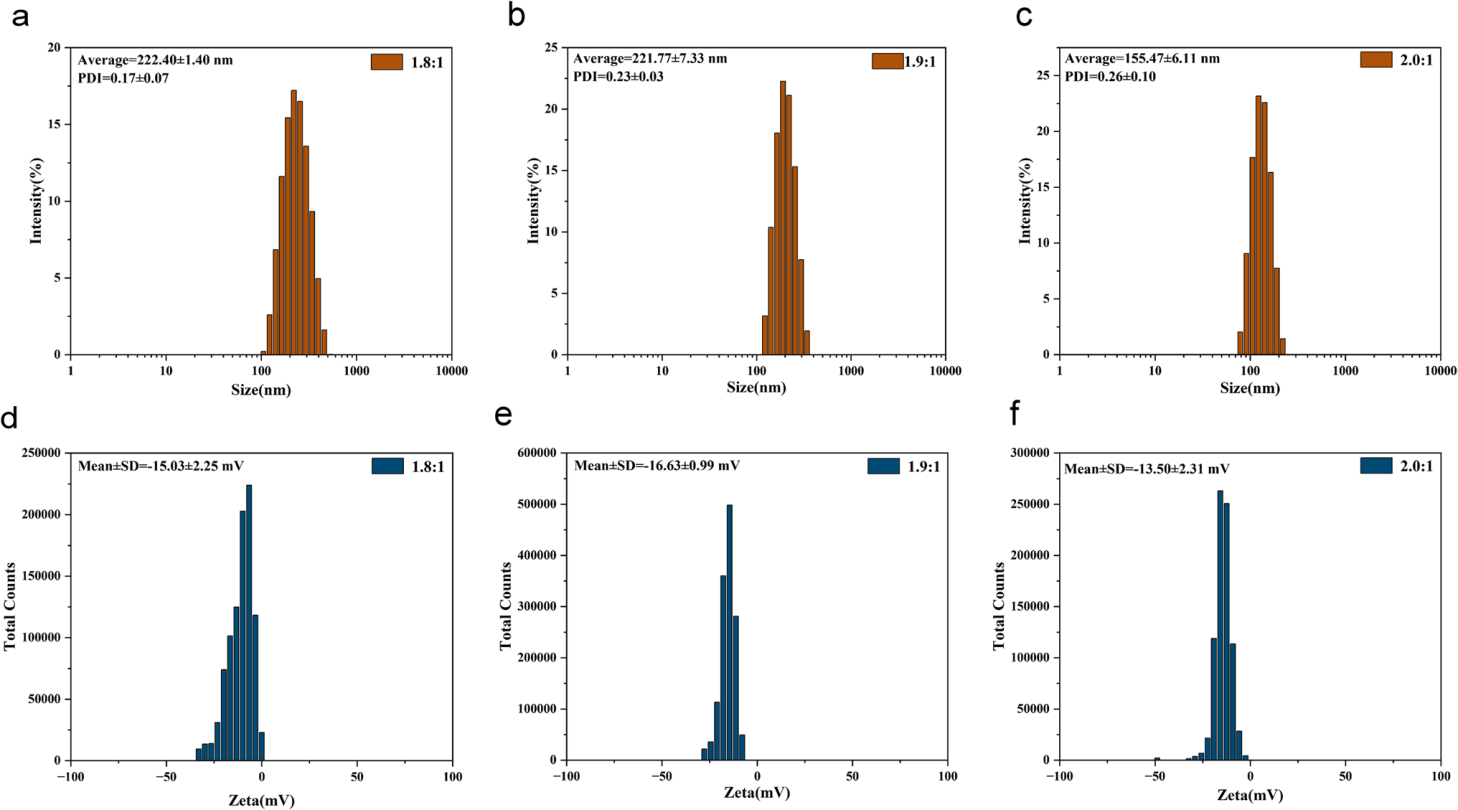
Characterization plots of the properties of HA-ANI NPs. **a** Particle size diagram for HA:ANI = 1.8:1. **b** Particle size diagram for HA:ANI = 1.9:1. **c** Particle size diagram for HA:ANI = 2.0:1. **d** Zeta potential diagram for HA:ANI = 1.8:1. **e** Zeta potential diagram for HA:ANI = 1.9:1. **f** Zeta potential diagram for HA:ANI = 2.0:1 graphs

**Fig. 4:**
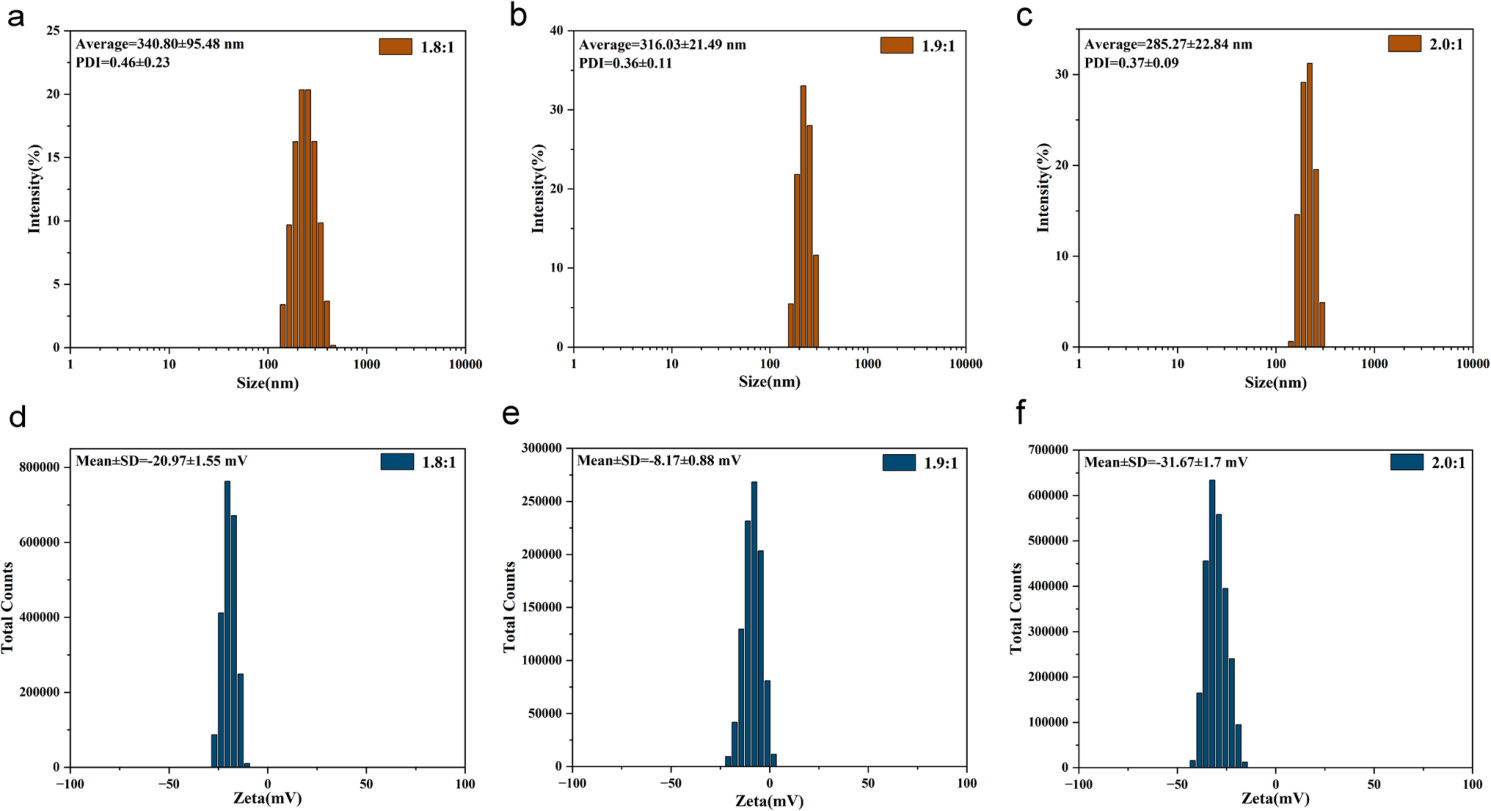
Characterization plots of the properties of P/G NPs. a Particle size plot for HA:ANI = 1.8:1. **b** Particle size plot for HA:ANI = 1.9:1. **c** Particle size plot for HA:ANI = 2.0:1. **d** Zeta potential plot for HA:ANI = 1.8:1. **e** Zeta potential plot for HA:ANI = 1.9:1. **f** Zeta potential plot for HA:ANI = 2.0:1 graphs

The average hydrodynamic diameter, zeta potential and PDI of blank nanoparticles and drug-loaded nanoparticles were measured by the dynamic light scattering method (Figs. 3 and 4), and the specific values of size, potential and PDI of blank nanoparticles and drug-loaded nanoparticles with different mass ratios of HA and ANI are listed in Tables 1 and 2, respectively. When HA:ANI = 2.0:1, the nanoparticles showed a better size distribution, the dispersion index (PDI) was < 0.3 for all ratios. Moreover, the PDI increased after drug loading, probably due to nanoparticle aggregation. All three ratios of nanoparticles have negative potential, which can ensure the stability of the nanoparticles in blood circulation.

**Table 1:** Comparison of the size, potential and PDI of nanoparticles with different ratios of HA-ANI NPs

HA:ANI	Size (nm)	Zeta (mV)	PDI
1.8:1	222.40 ± 1.40	− 15.03 ± 2.25	0.17 ± 0.07
1.9:1	221.77 ± 7.33	− 16.63 ± 0.99	0.23 ± 0.03
2.0:1	155.47 ± 6.11	− 13.50 ± 2.31	0.26 ± 0.10

**Table 2:** Comparison of nanoparticle size, potential and PDI for different ratios of P/G NPs

HA:ANI	Size (nm)	Zeta (mV)	PDI	C_PTX_ (μg/mL)	C_GEM_ (μg/mL)
1.8:1	340.80 ± 95.48	− 20.97 ± 1.55	0.46 ± 0.23	35.26	3.63
1.9:1	316.03 ± 21.49	− 8.17 ± 0.88	0.36 ± 0.11	26.35	8.36
2.0:1	285.27 ± 22.84	− 31.67 ± 1.76	0.37 ± 0.09	28.17	3.09

**Fig. 5:**
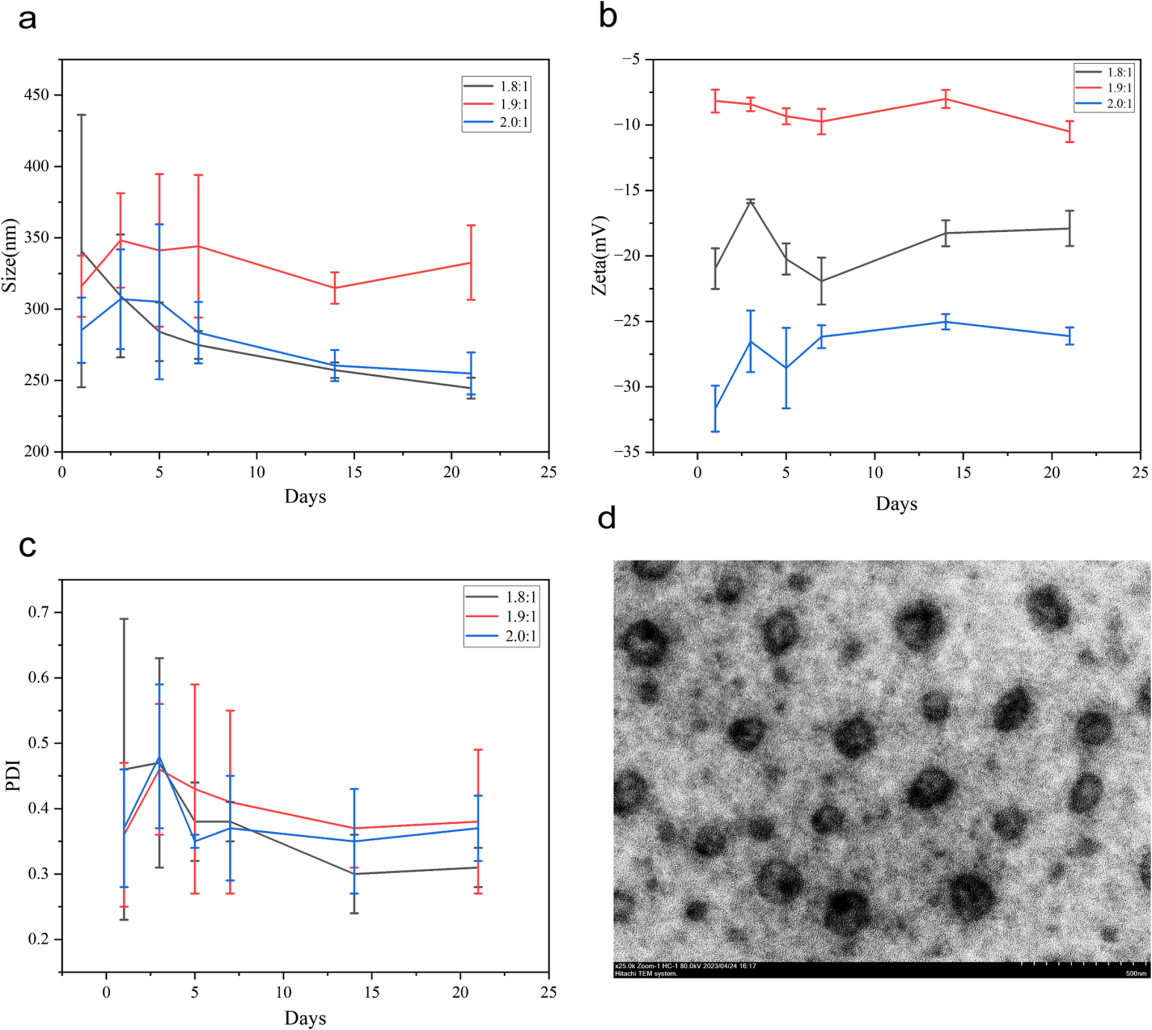
Characterization of P/G NP properties. **a** Particle size variation over 21 d for three ratios of P/G NPs (1.8:1, 1.9:1, 2.0:1). **b** Zeta potential variation over 21 d for three ratios of P/G NPs (1.8:1, 1.9:1, 2.0:1). **c** PDI variation over 21 d for three ratios of P/G NPs (1.8:1, 1.9:1, 2.0:1). **d** transmission electron microscopy images of P/G NPs with HA:ANI = 2.0:1

The morphology of P/G NPs with a ratio of 2.0:1 was observed by TEM (Fig. 5d), and it was found to show a subspherical shape and a more uniform distribution. Then, the stability conditions of the three ratios of drug-loaded nanoparticles stored at room temperature for 21 d were monitored (Fig. 5a, b and c), and it was found that the particle size of the P/G NPs varied more with HA:ANI = 1.8:1, which was close to 100 nm, and less with HA:ANI = 1.9:1 and 2.0:1, which was within 20 ~ 50 nm with a smaller PDI.

**Fig. 6:**
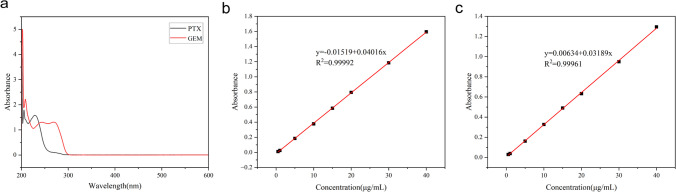
Establishment of PTX and GEM content determination methods. **a** Scanning spectra of PTX and GEM. **b** standard curve of PTX. **c** standard curve of GEM

In addition, the content of paclitaxel and gemcitabine was determined by UV spectrophotometry, and it was found that PTX had a maximum absorption at 229 nm and GEM had a maximum absorption at 269 nm by wave scan (Fig. 6a). The standard curve for PTX was made at 229 nm as Y = -0.01519 + 0.04016X (Fig. 6b), and the standard curve for GEM was made at 269 nm as Y = 0.00634 + 0.03189X (Fig. 6c). Then, we calculated the drug loading and encapsulation rate of the two drugs in P/G NPs using the standard curve, which are shown in Table 3. It was found that the nanoparticles had higher drug loading and encapsulation rates at HA:ANI = 2.0:1, with drug loading rates of 4.66% and 0.51% for PTX and GEM, and encapsulation efficiency of 12.68% and 1.30%, respectively. We used this ratio of nanoparticles for the follow-up study.

**Table 3:** P/G NP drug loading capacity and encapsulation efficiency

HA:ANI	PTX		GEM	
	LC (%)	EE (%)	LC (%)	EE (%)
1.8:1	3.05	13.22	0.21	1.27
1.9:1	3.75	7.38	0.54	2.39
2.0:1	4.66	12.68	0.51	1.30

### In vitro drug release studies

**Fig. 7:**
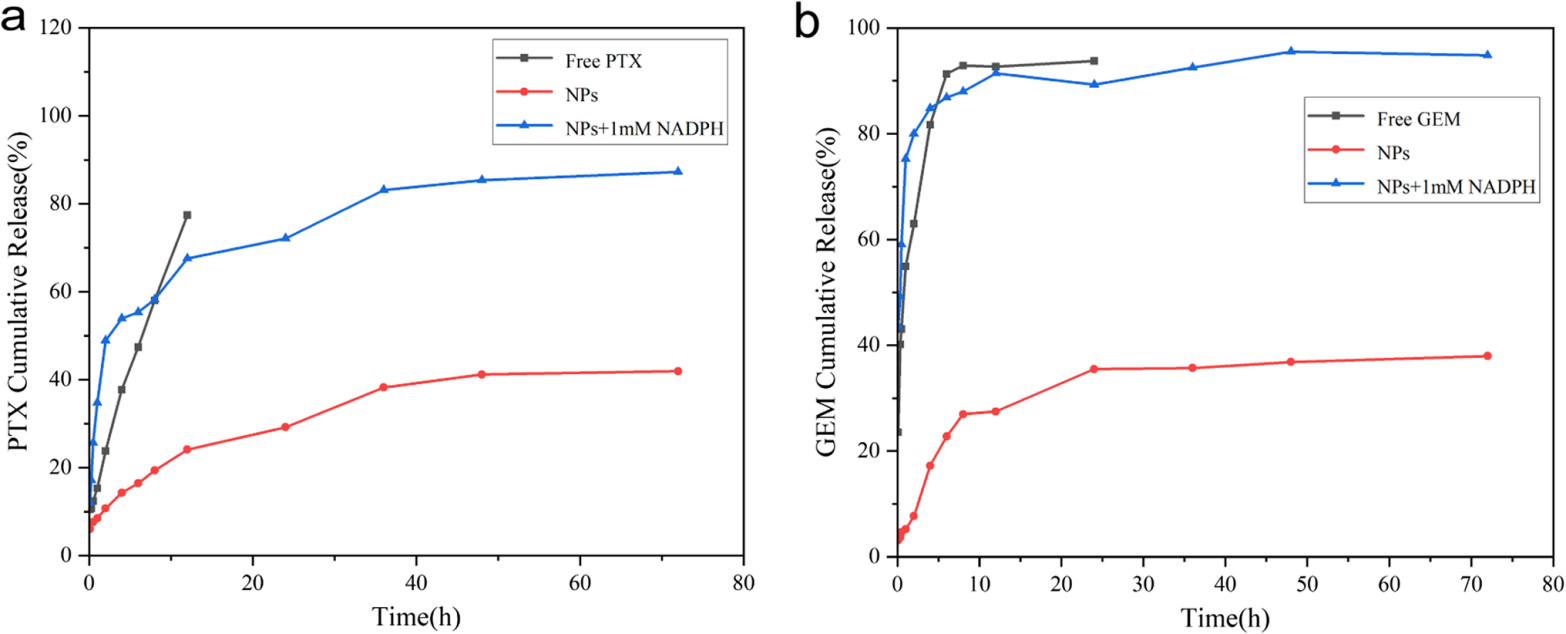
In vitro release curves of paclitaxel and gemcitabine in P/G NPs. **a** In vitro release profile of PTX. **b** In vitro release profile of GEM

We further tested the effect of PTX and GEM release from NPs in different release media in comparison with free PTX and GEM. As shown in Fig. 7a, free paclitaxel reached 77.42% release at 12 h in pH 7.4 buffer, drug-loaded nanoparticles reached 41.95% release at 72 h in pH 7.4 buffer, and nanoparticles with 1 mM NADPH added reached 87.28% release at 72 h. As shown in Fig. 7b, the release rate of free gemcitabine in pH 7.4 buffer reached 93.79% at 24 h. The drug-loaded nanoparticles in pH 7.4 buffer reached 37.97% at 72 h, while the nanoparticles with 1 mM NADPH added reached 94.83% at 72 h. We found that the release rate of nanoparticles increased significantly after the addition of NADPH. We speculate that this may be due to the reduction of nitro to amino caused by high levels of NADPH, further leading to the dissociation of the nanoparticles to release the drug.

### Cellular uptake

**Fig. 8:**
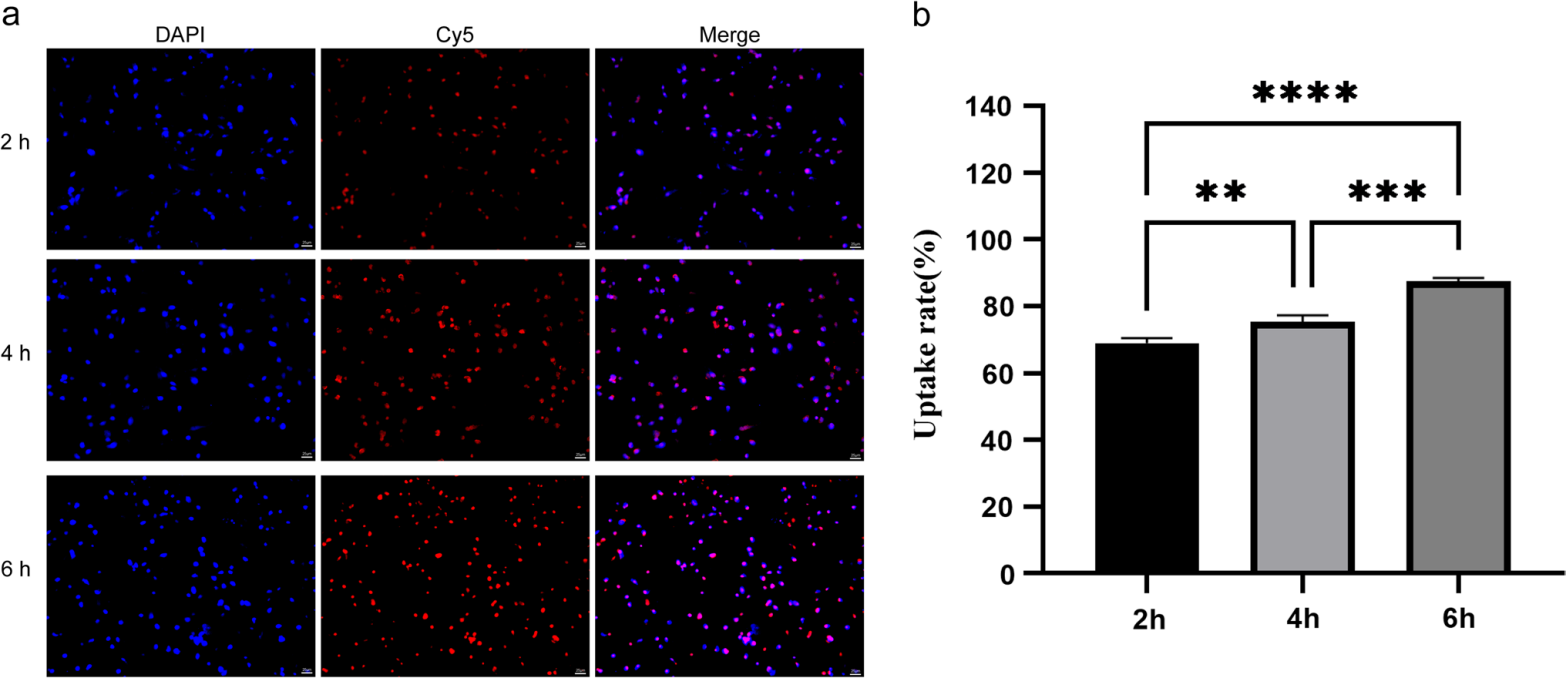
Cellular uptake analysis of Cy5 NPs. **a** Fluorescence images of MDA-MB-231 cells stained with DAPI and Cy5 NPs. **b** Statistical analysis of nanoparticle uptake rate

The uptake efficiency of NPs by MDA-MB-231 cells was analyzed by inverted fluorescence microscopy. As shown in Fig. 8a, Cy5 loaded within NPs showed red fluorescence, and DAPI stained cell nuclei and showed blue fluorescence. As shown in Fig. 8b, the quantitative analysis of the fluorescence of uptake revealed that the uptake rate was positively correlated with time, and the cellular uptake rate of NPs reached 86.50% at a coincubation time of 6 h with NPs, which was significantly higher than 69.10% at 2 h and 75.80% at 4 h. We suggest that the uptake time may affect the uptake rate of cells.

### Cell viability

**Fig. 9:**
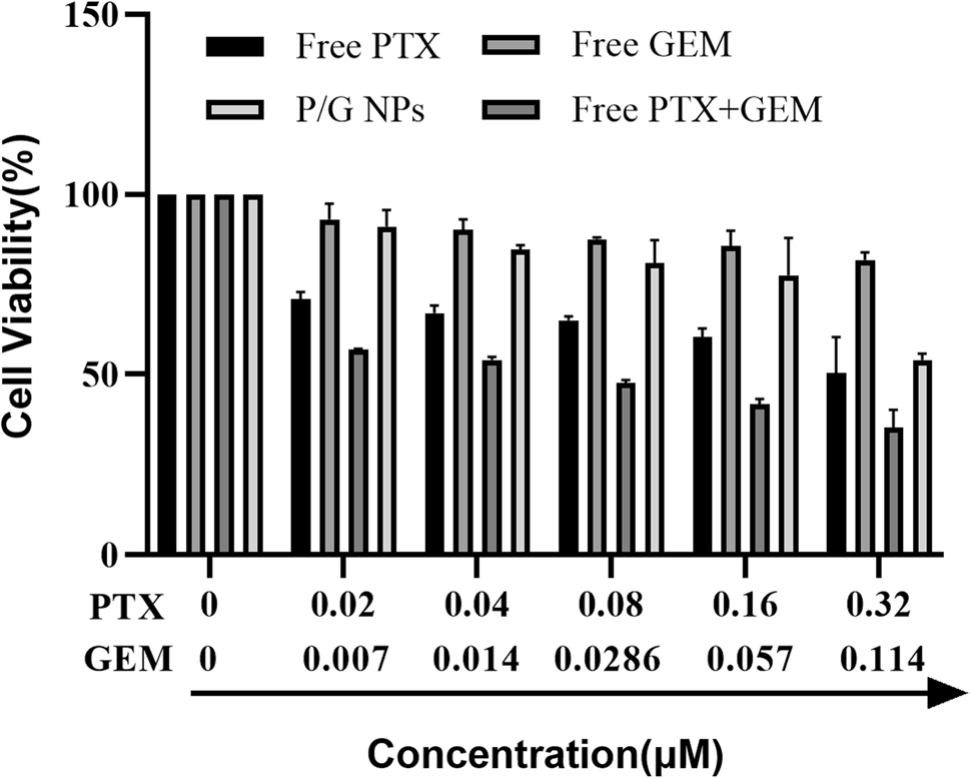
Cell viability of MDA-MB-231 cells after 48 h of treatment with different drug groups (free PTX, free GEM, free PTX + GEM and P/G NPs)

The toxic effects of different preparations (free PTX, free GEM, free PTX + GEM and P/G NPs) on MDA-MB-231 cells were assessed by the MTT method. As shown in Fig. 9, the IC50 values of free PTX, free GEM, free PTX + GEM and P/G NPs after incubation with cells for 48 h were 0.46 µM, 9.66 µM, 0.06 µM and 0.48 µM, respectively. At a PTX concentration of 0.32 µM, the toxic effect of P/G NPs on cells was comparable to that of free paclitaxel, at which time it was known from the release assay that the release rate of the nanoparticles had only reached approximately 80%; therefore, with the extension of the incubation time, it was speculated that stronger toxic effects might also be produced. All these results indicate that the codelivery of PTX and GEM with nanoparticles can significantly increase the toxic effects on cells compared to the use of either drug alone.

### Cell migration

**Fig. 10:**
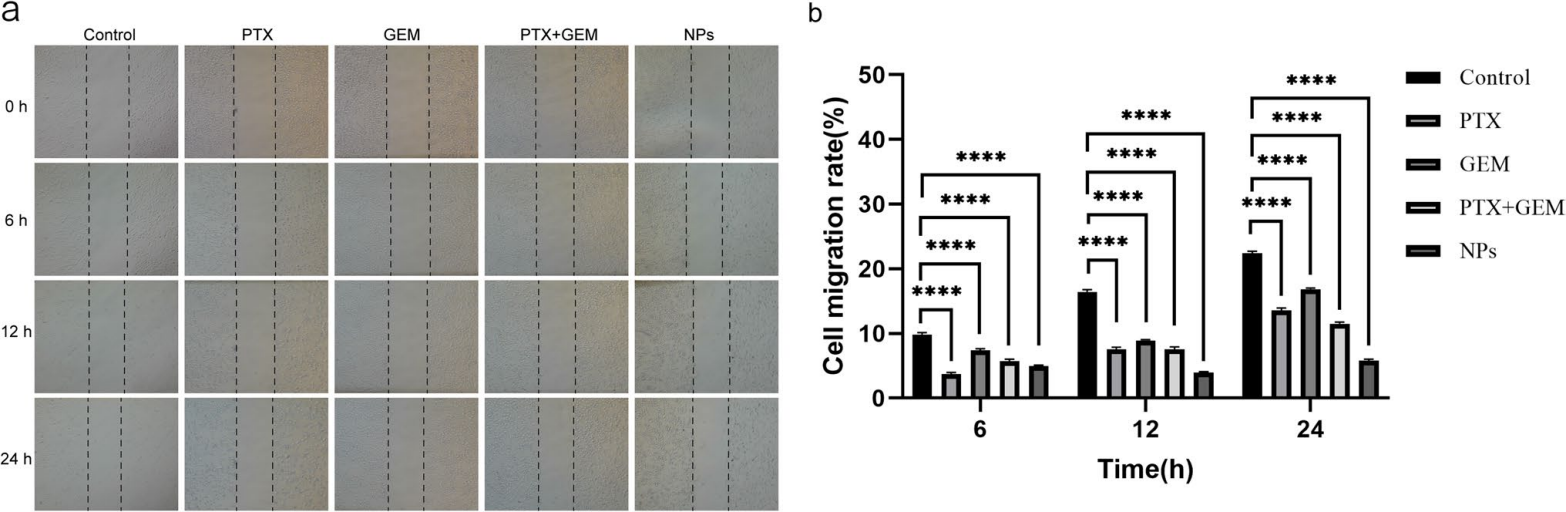
Cell migration experiments with different groups of drugs. **a** Images of the effects of different drug treatments on the migration of MDA-MB-231 cells. **b** Statistical analysis of migration rates

The cell scratch assay was used to evaluate the migration effect of cells in different drug treatment groups. As shown in Fig. 10, the cell migration rates at 24 h were 22.49%, 13.87%, 16.99%, 11.820 and 5.99% in the control, free PTX, free GEM, free PTX + GEM and P/G NP groups, respectively. The lowest cell migration rate was observed in the P/G NP group, which indicated that the nano-codelivery system could significantly enhance the combined PTX and GEM use on cell migration inhibition.

### Cell apoptosis assays

**Fig. 11:**
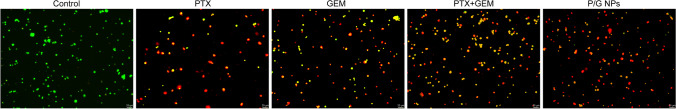
Results of AO&EB staining of MDA-MB-231 cells treated with different drug groups for 24 h

The AO/EB apoptosis assay kit was used to observe the effect of different drug treatment groups on apoptosis, as shown in Fig. 11. No obvious apoptosis was detected in the PBS group; some early apoptotic cells were observed in the free PTX, free GEM, free PTX + GEM, and P/G NP groups, marked by crescent-shaped or granular yellow‒green AO nuclear staining, and a large number of late apoptotic cells appeared in the P/G NP group, as indicated by concentrated and asymmetrically localized orange nuclear EB staining and necrotic cells. The volume increased and showed uneven orange‒red fluorescence around them. The degree of cell damage was significantly enhanced in the P/G NP group compared to the free group, indicating that the encapsulation of free drug by nanoparticles, at comparable concentrations, can significantly enhance the degree of apoptosis.

## Discussion

Since the combination of Abraxane and GEM demonstrates good synergistic effects in breast cancer [29], a single carrier was designed in this study to codeliver PTX and GEM with optimized carrier size and drug ratio to achieve the intended synergistic effect. HA-ANI nanocarriers were synthesized mainly by grafting hyaluronic acid with a nitroimidazole compound, and the amphiphilic polymer was self-assembled with PTX and GEM by hydrodialysis to form dual drug-carrying nanoparticles. The EPR effect of tumor cells makes the dual drug-loaded nanoparticles more easily enriched between tumor cells compared to normal cells. When the drug enters the tumor cells, the low oxygen microenvironment makes it extremely easy for the nanoparticles to reduce the nitro group in the carrier to an amino group under the action of the bioreductant and change from a hydrophobic group to a hydrophilic group. The nanoparticles dissociate and release PTX and GEM to exert antitumor effects. In conclusion, we prepared a nanocarrier for the efficient delivery of the dual drug PTX and GEM for the dual drug synergistic treatment of TNBC.

Many experimental methods have been developed for drug codelivery by nanocarriers, including direct encapsulation of drugs inside nanoparticles, covalent coupling of drug combinations to carriers, and surface attachment of drugs to existing drug carriers [30–32]. There are studies on liposomes encapsulating cytarabine and erythromycin at a molar ratio of 5:1 in a mouse model for the synergistic treatment of leukemia [33]; independent coupling of Adriamycin and camptothecin with particles having a polymeric backbone for the treatment of human breast cancer [34]; and loading of GEM monophosphate and cisplatin into PLGA particles at a 120:5 molar ratio for the treatment of bladder cancer [35].

In this study, we successfully prepared dual drug-loaded nanocarriers with nanoparticles of different sizes by adjusting the different ratios of hydrophilic and hydrophobic components in the carrier materials. We found that as the proportion of hydrophilic materials increased, the size decreased, and the drug loading and encapsulation rates increased. The results of in vitro release experiments of P/G NPs showed that the release rate of PTX increased from 41.95% to 87.28% at 72 h after the addition of 1 mM NADPH; the release rate of GEM increased from 37.97% to 94.83% at 72 h, which suggests that our nanocarriers may be dissociated by the action of NAPDH, resulting in rapid drug release. The investigation of the toxic effects of free drugs and nanoparticles on MDA-MB-231 cells showed that the cytotoxicity of the nano-group was significantly stronger than that of the free single drug group after 48 h of drug treatment. Although the direct combination of free drugs showed significant cytotoxic effects, it may lead to cumulative toxic effects. Therefore, NPs have significant advantages in terms of drug delivery methods and efficacy. The AO/EB double-staining assay observed that the P/G NPs group had the strongest cell damage effect after 24 h of drug treatment. The migration inhibition effect of different drug groups on cells was observed, and it was found that the cell migration rate of the nano-group was 5.99% at 24 h, much lower than the 13.87% and 17.00% of the free group. The aim of this study was to design a nanolocalized release formulation to perform the efficient delivery of dual drugs for localized drug release at the cellular level to exert a synergistic anti-TNBC effect. The nano-codelivery system in this study integrates good biocompatibility, environmental responsiveness, targeting, high stability and low toxicity into a single platform to provide a new therapeutic strategy against TNBC.

## Conclusion

In this study, nanoparticles with different ratios of hydrophilic and hydrophobic materials were prepared. The size of the nanoparticles decreased and the drug loading capacity increased as the proportion of the hydrophilic material HA was increased. Nanoparticles with a size distribution of 100–400 nm could be effectively taken up by MDA-MB-231 cells and exert strong inhibitory effects on cell growth and migration.

## Data Availability

The datasets generated during and/or analyzed during the current study are available from the corresponding author on reasonable request.

## Code availability

Source code is available upon request from the corresponding author.

## References

[1] Li Y, Zhang H, Merkher Y, Chen L, Liu N, Leonov S, Chen Y (2022). Recent advances in therapeutic strategies for triple-negative breast cancer. J Hematol Oncol.

[2] So JY, Ohm J, Lipkowitz S, Yang L (2022). Triple negative breast cancer (TNBC): Non-genetic tumor heterogeneity and immune microenvironment: emerging treatment options. Pharmacol Ther..

[3] Vagia E, Mahalingam D, Cristofanilli M (2020). The landscape of targeted therapies in TNBC. Cancers (Basel)..

[4] Qayoom H, Wani NA, Alshehri B, Mir MA (2021). An insight into the cancer stem cell survival pathways involved in chemoresistance in triple-negative breast cancer. Future Oncol.

[5] Manjunath M, Choudhary B (2021). Triple-negative breast cancer: a run-through of features, classification and current therapies. Oncol Lett.

[6] Kabraji S, Solé X, Huang Y, Bango C, Bowden M, Bardia A, Sgroi D, Loda M, Ramaswamy S (2017). AKT1(low) quiescent cancer cells persist after neoadjuvant chemotherapy in triple negative breast cancer. Breast Cancer Res.

[7] Ferrari P, Scatena C, Ghilli M, Bargagna I, Lorenzini G, Nicolini A (2022). Molecular mechanisms, biomarkers and emerging therapies for chemotherapy resistant TNBC. Int J Mol Sci.

[8] Abu Samaan TM, Samec M, Liskova A, Kubatka P, Büsselberg D (2019). Paclitaxel's mechanistic and clinical effects on breast cancer. Biomolecules.

[9] Yang CH, Horwitz SB (2017). Taxol(®): the first microtubule stabilizing agent. Int J Mol Sci.

[10] Yu DL, Lou ZP, Ma FY, Najafi M (2022). The interactions of paclitaxel with tumour microenvironment. Int Immunopharmacol.

[11] Wang YT, Li B, Li XG, Ma SK, Zhang R, Wu LY (2019). Efficacy and side effect analysis of paclitaxel liposome for neoadjuvant chemotherapy in locally advanced cervical cancer. Zhonghua Fu Chan Ke Za Zhi.

[12] Alavi M, Nokhodchi A (2022). Micro- and nanoformulations of paclitaxel based on micelles, liposomes, cubosomes, and lipid nanoparticles: Recent advances and challenges. Drug Discov Today.

[13] Pandit B, Royzen M (2022). Recent development of prodrugs of gemcitabine. Genes (Basel).

[14] Hu XC, Zhang J, Xu BH, Cai L, Ragaz J, Wang ZH, Wang BY, Teng YE, Tong ZS, Pan YY, Yin YM, Wu CP, Jiang ZF, Wang XJ, Lou GY, Liu DG, Feng JF, Luo JF, Sun K, Gu YJ, Wu J, Shao ZM (2015). Cisplatin plus gemcitabine versus paclitaxel plus gemcitabine as first-line therapy for metastatic triple-negative breast cancer (CBCSG006): a randomised, open-label, multicentre, phase 3 trial. Lancet Oncol.

[15] Wang B, Sun T, Zhao Y, Wang S, Zhang J, Wang Z, Teng YE, Cai L, Yan M, Wang X, Jiang Z, Pan Y, Luo J, Shao Z, Wu J, Guo X, Hu X (2022). A randomized phase 3 trial of Gemcitabine or Nab-paclitaxel combined with cisPlatin as first-line treatment in patients with metastatic triple-negative breast cancer. Nat Commun.

[16] Liu MC, Janni W, Georgoulias V, Yardley DA, Harbeck N, Wei X, McGovern D, Beck R (2019). First-line doublet chemotherapy for metastatic triple-negative breast cancer: circulating tumor cell analysis of the tnAcity trial. Cancer Manag Res.

[17] Zhou L, Zou M, Xu Y, Lin P, Lei C, Xia X (2022). Nano drug delivery system for tumor immunotherapy: next-generation therapeutics. Front Oncol.

[18] Li B, Shao H, Gao L, Li H, Sheng H, Zhu L (2022). Nano-drug co-delivery system of natural active ingredients and chemotherapy drugs for cancer treatment: a review. Drug Deliv.

[19] Qiu Z, Yu Z, Xu T, Wang L, Meng N, Jin H, Xu B (2022). Novel nano-drug delivery system for brain tumor treatment. Cells.

[20] Wang C, Li F, Zhang T, Yu M, Sun Y (2022). Recent advances in anti-multidrug resistance for nano-drug delivery system. Drug Deliv.

[21] Zhang Y, Zhang W, Wang Y, Zhu J, Zhou M, Peng C, He Z, Sun J, Li Z, Gui S (2021). Emerging nanotaxanes for cancer therapy. Biomaterials.

[22] Zhang N, Feng N, Xin X, Zhang J, Wu D, Jiang Q, Yu T, Gao M, Zhao S, Yang H, Tian Q (2022). Nano-drug delivery system with enhanced tumour penetration and layered anti-tumour efficacy. Nanomedicine.

[23] Kearns O, Camisasca A, Giordani S (2021). Hyaluronic acid-conjugated carbon nanomaterials for enhanced tumour targeting ability. Molecules.

[24] Jahedi M, Meshkini A (2023). Tumor tropic delivery of FU.FA@NSs using mesenchymal stem cells for synergistic chemo-photodynamic therapy of colorectal cancer. Colloids Surf B Biointerfaces..

[25] Gao Y, Qiu W, Liang M, Ma X, Ye M, Xue P, Kang Y, Deng J, Xu Z (2022). Active targeting redox-responsive mannosylated prodrug nanocolloids promote tumor recognition and cell internalization for enhanced colon cancer chemotherapy. Acta Biomater.

[26] Ding H, Tan P, Fu S, Tian X, Zhang H, Ma X, Gu Z, Luo K (2022). Preparation and application of pH-responsive drug delivery systems. J Control Release.

[27] Xiao Y, Zhang T, Ma X, Yang QC, Yang LL, Yang SC, Liang M, Xu Z, Sun ZJ (2021). Microenvironment-responsive prodrug-induced pyroptosis boosts cancer immunotherapy. Adv Sci (Weinh).

[28] Yu H, Jin F, Liu D, Shu G, Wang X, Qi J, Sun M, Yang P, Jiang S, Ying X, Du Y (2020). ROS-responsive nano-drug delivery system combining mitochondria-targeting ceria nanoparticles with atorvastatin for acute kidney injury. Theranostics.

[29] Delfino C, Caccia G, Riva Gonzáles L, Mickiewicz E, Rodger J, Balbiani L, Flores Morales D, Zori Comba A, Brosio C (2003). Gemcitabine/paclitaxel as first-line treatment of advanced breast cancer. Oncology (Williston Park).

[30] Lin T, Zhao P, Jiang Y, Tang Y, Jin H, Pan Z, He H, Yang VC, Huang Y (2016). Blood-brain-barrier-penetrating albumin nanoparticles for biomimetic drug delivery via albumin-binding protein pathways for antiglioma therapy. ACS Nano.

[31] Irby D, Du C, Li F (2017). Lipid-drug conjugate for enhancing drug delivery. Mol Pharm.

[32] Pérez-Herrero E, Fernández-Medarde A (2015). Advanced targeted therapies in cancer: drug nanocarriers, the future of chemotherapy. Eur J Pharm Biopharm.

[33] Tardi P, Johnstone S, Harasym N, Xie S, Harasym T, Zisman N, Harvie P, Bermudes D, Mayer L (2009). In vivo maintenance of synergistic cytarabine:daunorubicin ratios greatly enhances therapeutic efficacy. Leuk Res.

[34] Aryal S, Hu CM, Zhang L (2011). Polymeric nanoparticles with precise ratiometric control over drug loading for combination therapy. Mol Pharm.

[35] Miao L, Guo S, Zhang J, Kim WY, Huang L (2014). Nanoparticles with precise ratiometric co-loading and co-delivery of gemcitabine monophosphate and cisplatin for treatment of bladder cancer. Adv Funct Mater.

